# Identifying the Metabolic Differences of a Fast-Growth Phenotype in *Synechococcus* UTEX 2973

**DOI:** 10.1038/srep41569

**Published:** 2017-01-31

**Authors:** Thomas J. Mueller, Justin L. Ungerer, Himadri B. Pakrasi, Costas D. Maranas

**Affiliations:** 1 Department of Chemical Engineering, Pennsylvania State University, University Park, Pennsylvania, USA; 2 Department of Biology, Washington University, St. Louis, Missouri, USA; 3 Department of Energy, Environmental, and Chemical Engineering, Washington University, St. Louis, Missouri, USA

## Abstract

The photosynthetic capabilities of cyanobacteria make them interesting candidates for industrial bioproduction. One obstacle to large-scale implementation of cyanobacteria is their limited growth rates as compared to industrial mainstays. *Synechococcus* UTEX 2973, a strain closely related to *Synechococcus* PCC 7942, was recently identified as having the fastest measured growth rate among cyanobacteria. To facilitate the development of 2973 as a model organism we developed in this study the genome-scale metabolic model *i*Syu683. Experimental data were used to define CO_2_ uptake rates as well as the biomass compositions for each strain. The inclusion of constraints based on experimental measurements of CO_2_ uptake resulted in a ratio of the growth rates of *Synechococcus* 2973 to *Synechococcus* 7942 of 2.03, which nearly recapitulates the *in vivo* growth rate ratio of 2.13. This identified the difference in carbon uptake rate as the main factor contributing to the divergent growth rates. Additionally four SNPs were identified as possible contributors to modified kinetic parameters of metabolic enzymes and candidates for further study. Comparisons against more established cyanobacterial strains identified a number of differences between the strains along with a correlation between the number of cytochrome c oxidase operons and heterotrophic or diazotrophic capabilities.

Cyanobacteria are a morphologically and geographically diverse group of photosynthetic prokaryotes that have adapted to a wide variety of environmental niches including fresh, sea, and wastewater^1^,^2^,^3^. These organisms therefore do not compete for arable land with food and energy crops^4^. Cyanobacteria have been explored as candidates for numerous applications, including carbon dioxide sequestration^5^,^6^ and as a food source^7^,^8^. In recent years, cyanobacteria have become the targets of synthetic biology for use as platforms for the production of a variety of industrially relevant chemicals^9^,^10^. These include both biochemically active products^11^,^12^, as well as a number of biofuel products such as hydrogen^13^, butanol^14^ and polymer precursors including 2,3-butanediol^15^,^16^ and isoprene^17^.

Despite the inherent advantage of not requiring a reduced carbon substrate, cyanobacteria are still limited by growth rates lower than industrial mainstays such as *E. coli* and *Saccharomyces cerevisiae.* The model cyanobacterium *Synechocystis* PCC 6803 has a maximum doubling time of 7–10 hours under optimal conditions^18^. However a recently discovered cyanobacterium, *Synechococcus* UTEX 2973 has been shown to have the lowest doubling time among cyanobacteria at 1.9 hours^19^. This organism is closely related to *Synechococcus* PCC 7942, with a total of 55 single nucleotide polymorphisms and insertion-deletions, including a 188.6 kb inversion and a deletion of six open reading frames that are present in 7942^19^. Despite the small differences, the growth rate for 2973 is 2.13 times greater than that of 7942 under their optimal illumination conditions at 38C^19^. This rapid growth rate and recent development of additional genetic tools^20^ makes *Synechococcus* 2973 an attractive candidate for bioproduction. Developing *Synechococcus* 2973 as a platform requires a more thorough understanding of its metabolic network and capabilities.

Genome-scale metabolic (GSM) models provide a blueprint for tracing all possible routes for utilizing carbon substrates in the context of the overall metabolism. They contain the set of metabolic reactions catalyzed within the organism, the gene-protein-reaction (GPR) relationships, and reaction directionalities^21^,^22^. These models also have a biomass equation, which contains the experimentally measured stoichiometric amounts of biomass precursors for the specific organism. GSM models are created using the sequenced genome for the organism along with available literature evidence. Semi-automated model generation balances the significant time investment required for *de novo* model development with the risk of losing details specific to certain organisms associated with completely automated development^23^. The workflow introduced and implemented in Mueller *et al*. uses an available curated model and reviewed gene annotations to expedite the development of a GSM model^23^. The GSM models required for semi-automated model development of cyanobacteria strains already exist, as models have been developed for a number of cyanobacteria including *Synechocystis* PCC 6803^24^,^25^, *Cyanothece* ATCC 51142^24^, and *Synechococcus* PCC 7002^26^.

Once a GSM model is created, flux balance analysis (FBA) can be used to estimate the maximum theoretical biomass yield by maximizing the flux through the biomass equation^27^. Flux variability analysis (FVA) can then be used to determine the feasible ranges for each reaction flux at maximum growth conditions. GSM models can also be used to predict differences in pathways between organisms^23^, product yields^28^ and the effect of genetic interventions^29^. GPR relationships enable the modeling of gene knockout phenotypes. This can be used not only for model refinement^30^ but also to facilitate organism design by suggesting genetic manipulations to reapportion fluxes to obtain a desired phenotype^29^.

In this paper we describe the development of a composite GSM model for both *Synechococcus* 7942 and *Synechococcus* 2973 using a previously defined workflow^23^, the sequenced genomes, and a curated reference GSM model^24^. The *i*Syn731 GSM model of *Synechocystis* PCC 6803 was chosen as the reference model following the identification of similar percent identity between previously modeled cyanobacteria^24^,^26^ and the *Synechococcus* strains (see Materials and Methods). These two strains are represented via a composite model given that the minimal genomic differences between the two strains do not result in any changes to the set of reactions in each strain. The model is combined with additional experimental measurements; specifically carbon dioxide uptake rates and biomass composition, to further interrogate what factors contribute to these differences in growth rates. When the two strains were evaluated, the same reaction network was used with carbon dioxide uptake rates and a biomass reaction specific to each strain. Additional model refinement was performed by comparing the model against available gene essentiality data^31^. Using experimental measurements for CO_2_ uptake as constraints on the model, the *in silico* ratio of the growth rate of *Synechococcus* 2973 to *Synechococcus* 7942 of 2.03 is close to the *in vivo* ratio of 2.13, identifying differing carbon uptake rates as the main contributing factor to the fast-growth phenotype. Specific SNPs and their possible effects on metabolism and the fast-growth growth phenotype are discussed. Genomic comparisons between the *Synechococcus* strains and several subsets of organisms identify genes and functionalities exclusive to the two strains.

## Results and Discussion

### Model Generation and Testing

This work resulted in the creation of a composite GSM model for two closely related cyanobacteria strains, *Synechococcus* PCC 7942 and *Synechococcus* UTEX 2973 (hereafter referred to as *Synechococcus* 7942 and *Synechococcus* 2973). The composite model contains 1,178 reactions and 1,028 metabolites. GPRs were developed for both strains, with the *Synechococcus* 2973 GPRs containing 683 genes and the *Synechococcus* 7942 GPRs containing 687 genes. This difference in gene number is due to four pairs of *Synechococcus* 7942 genes that each map to a single *Synechococcus* 2973 gene. The composite model contains 150 reactions not present in *i*Syn731^24^, the original *Synechocystis* model used in the model generation workflow. The energetic parameters from *i*Syn731 (i.e. non-growth and growth associated maintenance energy costs) were also used in the *Synechococcus* model. 145 reactions were not transferred from *i*Syn731 to the composite model. 93 of these reactions have a GPR and did not have the necessary homologs to transfer.

**Figure 1:**
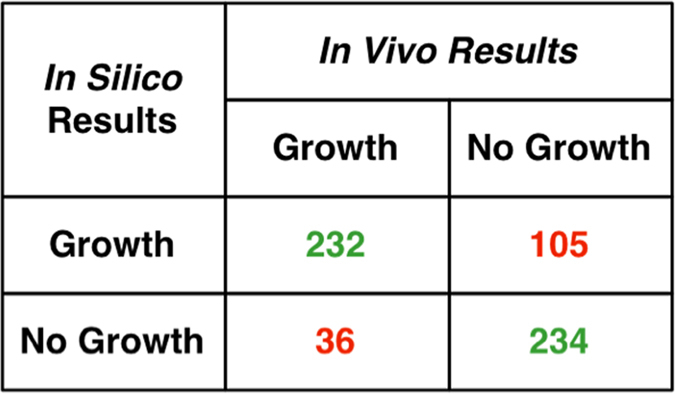
Categorization of *in silico* gene knockout predictions in *Synechococcus* 7942 as compared to *in vivo* data.

607 of the 687 genes in *i*Syu683 have *in vivo* essential/nonessential verdicts from the transposon library by Rubin *et al*.^31^. *In silico* predictions of these gene knockouts correctly predicted 232 of 268 non-essential genes and 234 of 395 essential genes (Fig. 1). All refinements made to the *Synechococcus* 7942 GPR were mapped to the *Synechococcus* 2973 GPR as well. Currently only one gene in the set of *Synechococcus* 2973 GPR relationships, *zwf,* has been knocked out. Both *in vivo* and *in silico* results indicate that *zwf*, which codes for glucose-6-phosphate 1-dehydrogenase in the oxidative pentose phosphate pathway, is not an essential gene for growth under autotrophic conditions (Abernathy *et al*., in review).

### Composition and Genomic Differences Between Strains

Measurements for each strain were obtained for amino acids, lipids, glycogen, and chlorophyll (Abernathy *et al*., in review) and used in the formulation of strain specific biomass equations (see Materials and Methods for a full description of biomass equation development). The most pronounced difference between the strains is in the glycogen levels, with *Synechococcus* 7942 generating 8.3 times the amount produced by *Synechococcus* 2973 during the exponential growth phase (Abernathy *et al*., in review). This supports the observations made by Yu *et al*. on the number of electron-dense bodies between the two strains^19^. Another significant difference is between amino acid levels, with 53.0% of *Synechococcus* 2973 biomass by weight composed of amino acids, as compared to 40.9% for *Synechococcus* 7942. Both strains have similar weight percentages of lipids, although measurements for C18:0 and C18:1 fatty acid chains are an order of magnitude higher in *Synechococcus* 7942. The weight percentage of chlorophyll a in the two strains is also quite similar, with 2973 having only 1.05 times the amount of the pigment found in 7942. These measured components sum to 755.46 and 743.89 mg/gDW for *Synechococcus* 7942 and *Synechococcus* 2973, respectively. Therefore, the unmeasured biomass components (e.g. nucleic acids) account for similar fraction of biomass.

Yu *et al*. compared the *Synechococcus* 7942 and 2973 genomes and identified 26 SNPs in 21 genes and six open reading frames (ORFs) present in *Synechococcus* 7942 that were not found in *Synechococcus* 2973. The six ORFs not found in *Synechococcus* 2973 are five hypothetical proteins and a chromophore lyase, none of which are present within the *Synechococcus* 7942 GPR. Of the 21 genes with SNPs, 11 are found within the *Synechococcus* 7942 GPR (Supplementary File S4). Seven of these genes are essential for carrying out 22 different reactions within the model. 12 of these reactions are associated with long-chain fatty acid CoA ligase. Nine of the ten remaining reactions can carry flux at experimental biomass, two of which are in nucleic acid synthesis and salvage pathways, two are ATP synthase reactions located at the thylakoid lumen and periplasm respectively, and five are in amino acid biosynthesis pathways (Table 1). SNPs in these genes could result in an increase to the kinetic parameters associated with the enzyme and contribute to a higher production of corresponding biomass components. When examining flux ranges standardized by carbon uptake for both strains at experimental growth rates, four ORFs with SNPs (M744_01335, M744_06650, M744_06850, and M744_12285) have associated reactions with higher achievable fluxes in *Synechococcus* 2973. This suggests that these locii code for enzymes that may have different kinetic constants between the two strains. This calls for enzymology studies of the enzymes with SNPs and kinetic modeling to assess the effect of kinetic parameter tune-up on growth rate.

**Figure 2:**
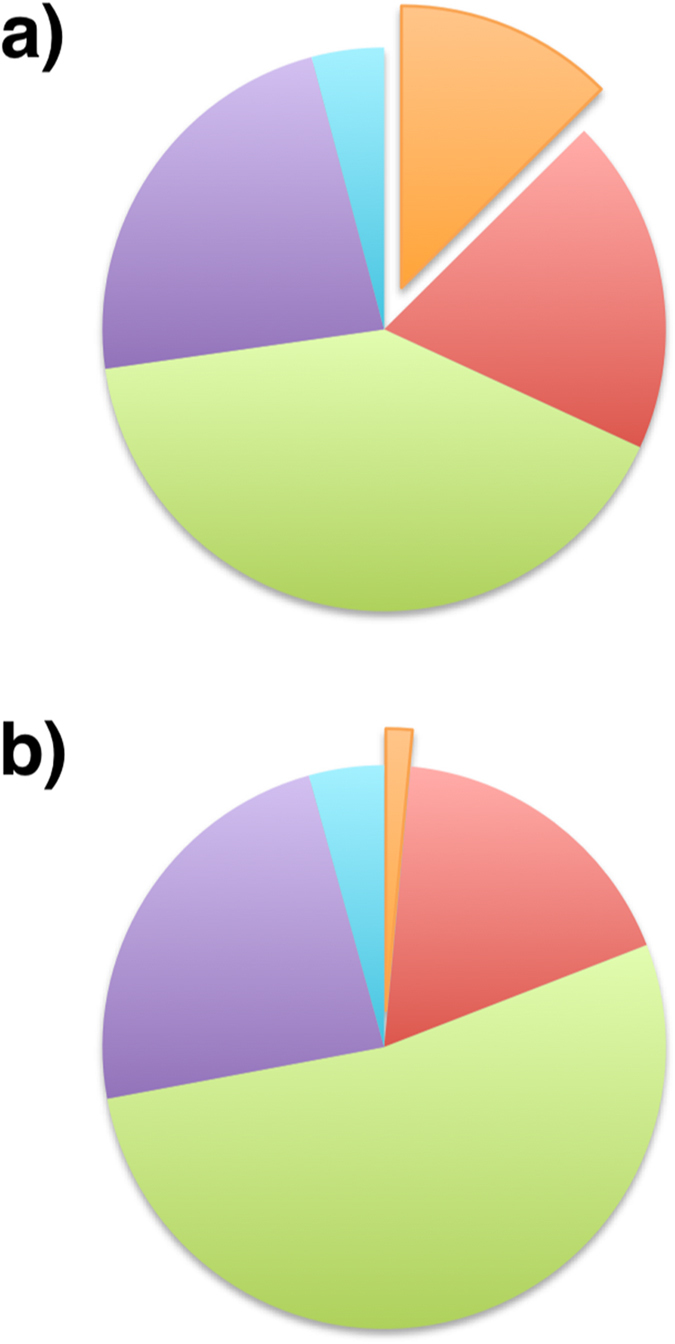
Mass fractions of the main constituents of biomass for the two *Synechococcus* strains. (**a**) Mass fractions for *Synechococcus* 7942 (**b**) Mass fractions for *Synechococcus* 2973. Color-Category relationship: orange – glycogen, red – lipids, green – amino acids, purple – nucleic acids, blue – other.

The remaining 10 genes with SNPs are not present in the GSM model and are associated with a diverse group of Gene Ontology (GO) categories^32^. Several SNPs imply potential effects that cannot be captured with a GSM model. Specifically, several SNP’s in RNA polymerase 23 S ribosomal RNA could result in higher rates of translation, which may explain the higher amino acid content in *Synechococcus* 2973 biomass as compared to *Synechococcus* 7942 (Fig. 2). Another SNP is in M744_13540, annotated as the Photosystem 1 assembly protein Ycf4. The *ycf4* knockout has been shown to be essential for PSI activity in *Chlamydomonas reinhardtii*^33^ and inactivation of an *ycf4* homolog in *Synechocystis* 6803 increases the PSII-PSI ratio^34^. Comparing the flux through the photosystem reactions under limiting light and maximum biomass production, *Synechococcus* 7942 has a higher PSII-PSI ratio than *Synechococcus* 2973, a difference that could correlate to an increased activity from M744_13540. While the effects of these changes cannot be captured with stoichiometric modeling they provide a starting point for further exploration with models that capture in detail more biological processes.

### Identification of Causes for Fast-Growth Phenotype

**Figure 3:**
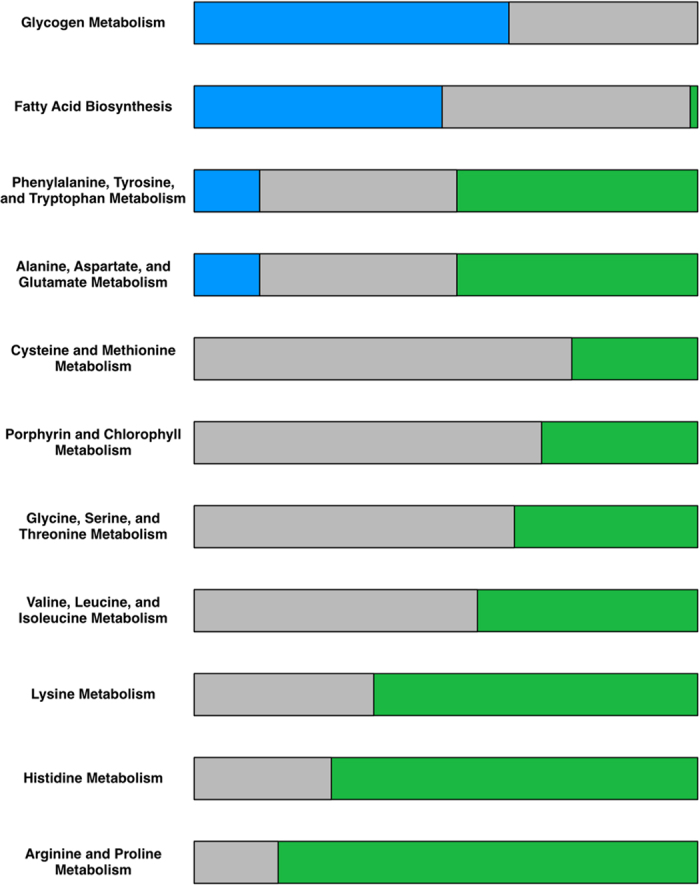
Fraction of select pathways with non-overlapping flux ranges between the two *Synechococcus* strains. Pathway annotations from iSyn731 and SEED were used. All flux ranges were standardized by the amount of carbon taken up. Grey represented the percentage of reactions with that pathway annotation whose flux ranges overlapped. Blue and green represent the percentage of reactions with non-overlapping ranges where *Synechococcus* 7942 and *Synechococcus* 2973 had higher flux respectively.

Under their optimal illumination conditions at 38 C, the growth rate ratio of *Synechococcus* 2973: *Synechococcus* 7942 is 2.13^19^. The GSM model can aid in the identification of a set of contributing factors to this difference. The varying biomass composition for the two strains and constant growth, transport, and maintenance energy requirements implies different biomass yields per mmol of CO_2_ taken up. The required moles of carbon for one theoretical mole of biomass were calculated by multiplying biomass stoichiometric coefficients by the number of carbon atoms in each precursor. *Synechococcus* 2973 requires 0.502 g carbon per g biomass as compared to 0.497 for *Synechococcus* 7942. When supplied equal amounts of CO_2_ the ratio of biomass yields per mol of carbon of *Synechococcus* 2973 to *Synechococcus* 7942 is 0.98 implying that *Synechococcus* 2973 requires slightly more carbon per gDW of biomass. Experimental measurements have shown that under their optimal conditions, *Synechococcus* 2973 takes up CO_2_ at a rate 2.06 times that of 7942 (Supplementary File S5). Using these measurements as a constraint on the exchange fluxes on the model resulted in an *in silico* growth rate ratio of 2.03. This is close to recapitulating the experimental ratio of 2.13. Therefore, due to the carbon limitation of the models, the difference in the rates of CO2 uptake is a primary contributing factor to the different growth rates seen for the two *Synechoccocus* strain models. The minimum required photons for both strains were calculated by minimizing the sum of photons taken up for both photosystems at maximum biomass. Flux variability analysis (FVA) was used to calculate the flux ranges for both strains at max biomass with minimum required photons. When the flux ranges are standardized by the amount of CO_2_ taken up the reactions with non-overlapping ranges are dictated by the biomass composition, with 2973 having a higher flux through the amino acid biosynthetic pathways and 7942 having a higher flux through the biosynthesis pathways for glycogen as well as C18:0 and 18:1 lipids (Fig. 3). Other notable differences include the aforementioned ratio of photon use between photosystems 1 and 2, and a higher flux through photorespiration in *Synechococcus* 2973. Photorespiration, the photosynthetic oxygenation of ribulose 1,5-bisphosphate (RuBP), competes with the carboxylation of RuBP and generates 2-phosphoglycolate (2PG). Eisenhut *et al*. previously showed that 2PG metabolism is essential for organisms such as cyanobacteria that perform oxygenic photosynthesis^35^, and similar requirements for flux through the photorespiration reaction at optimal growth rates has been seen in other *in silico* models^36^. The higher flux through photorespiration in *Synechococcus* 2973 could be due to the production of glyoxylate, a precursor to glycine, serine, and cysteine, from the photorespiration pathway and the increased requirements for amino acids in *Synechococcus* 2973.

With the supplied CO_2_ flux, both models can achieve growth rates higher than experimentally reported, with *Synechococcus* 2973 achieving a growth rate of 0.4569 hr^−1^ (doubling time: 1.5 hours) as compared to its experimentally reported optimal rate of 0.3014 hr^−1^ (doubling time: 2.3 hours), and *Synechococcus* 7942 with a growth rate of 0.2256 hr^−1^ (doubling time: 3.1 hours) as compared to the experimental value of 0.1415 hr^−1^ (doubling time: 4.9 hours). When the flux through the biomass reaction was fixed to the experimentally reported values, the model identified the main destinations of excess carbon as TCA cycle intermediates or metabolites that are several reactions removed from the TCA cycle (i.e. pyruvate, acetate, and glutamate). In total 33.4% of the carbon taken up is exported for 2973 and 37.2% is exported for 7942. This increased fraction of carbon allocated to biomass in 2973 could in part contribute to the divergent growth rates.

### Comparing *Synechococcus* with Model Organisms

Given *Synechococcus* 2973’s potential as a production strain, it is important to compare its capabilities with that of model cyanobacteria, with pairwise genome-wide BLAST comparisons performed between *Synechococcus* 7942 and *Synechocystis* PCC 6803, *Synechococcus* PCC 7002, Cyanothece ATCC 51142, *Anabaena* PCC 7120, and *Anabaena variabilis*. A set of 696 genes in 7942 does not have satisfactory hits in the other five genomes (See Materials and Methods for BLAST hit criteria). 47 of these genes are in the 7942 GPR relationships and are essential for 21 reactions that can carry flux at max biomass production. These reactions are spread across a variety of pathways including amino acid and deoxyribonucleotide biosynthesis. As these genes comprise only 6.8% of the genes within the *Synechococcus* 7942 GPR relationships, a majority of the metabolic genes have homologs in other cyanobacterial strains. Therefore insights gained into the metabolism of these strains can more readily inform work in the metabolism of the fast-growing strain. Expanding past metabolism, gene ontology (GO) terms provide a means to contextualize the remaining genes. Of these 696 genes, 304 have annotations including GO terms in the Uniprot database. This fraction of genes with GO terms (43.7%) is much lower than that of the entire 7942 genome (70.7%), following a trend seen in the percent of reviewed annotations (3.3% in the subset compared to 18.1% overall) and percent of genes annotated as uncharacterized (70.4% in the subset and 39.2% overall). Two GO terms have at least ten genes in the subset and greater than 50% of the genes in the organism with that GO term within the subset. There are 14 genes within this subset annotated with the term GO:0000155 – phosphorelay sensor kinase activity, and 10 genes annotated with the term GO:0043565 – sequence specific DNA binding. This suggests that most of the differences between these strains are related to the response to external stimuli and transcription regulation. These differences are consistent with the comparison of a number of thioredoxins involved in light dependent protein regulation^37^. The BLAST search identified three homologs in *Synechococcus* 7942 in contrast to four and eight thioredoxin genes in *Synechocystis* 6803 and *Anabaena* 7120^38^.

Another key difference between cyanobacterial strains is the classification of some organisms as obligate photoautotrophs. Schmetterer *et al*. showed that the deletion of a three gene cytochrome c oxidase (*coxBAC*) operon from *Anabaena variabilis* ATCC 29413 resulted in a strain capable of unhindered photoautotrophic growth but which was no longer capable of chemoheterotrophic growth^39^. BLAST searches were performed between *A. variabilis* 29413’s five operons and seven other cyanobacteria with information on their ability to grow heterotrophically. An independent annotation search of each organism was also performed, which identified the same operon sets for each organism as were identified through the BLAST search. Table 2 shows the results of these comparisons, with obligate photoautotrophs all having a single operon, as compared to two or more for each organism capable of heterotrophic growth. However, organisms capable of nitrogen fixation have at least three operons while the non-diazotrophic heterotrophs are restricted to two operons. Several specialized roles have been identified for specific *coxBAC* operons including an operon required for growth on sugar^39^ and separate operons that are heterocyst specific and required for nitrogen fixation^40^. These roles suggest a possible cause for the varying number of operons across cyanobacterial strains, and provide a promising avenue for further comparison among cyanobacteria.

## Conclusions

In this paper we presented a GSM model for both *Synechococcus* 7942 and *Synechococcus* 2973 with GPR relationships that were tested and refined using gene essentiality data for *Synechococcus* 7942. This model was used to compare the two strains and identify possible reasons for the diverging fast-growth phenotype. The GSM model allowed for the identification of the pathways where flux was allocated to differing levels between the two strains along with the identification of four SNPs as possible contributors to modified kinetic parameters of metabolic enzymes and candidates for further study. Several differences between the two strains were identified including the fraction of carbon allocated to biomass and the weight fractions of different biomass precursors, with the most prominent being the difference in carbon uptake rates, which contributed to the different *in silico* growth rates. Finally, a genomic comparison between *Synechoccocus* 7942 and model organisms highlighted a number of differences including regulatory genes without identified homologs and a variation in the number of copies of the cytochrome c oxidase operon as related to heterotrophic and diazotrophic capabilities.

## Methods

### GSM Model Generation

The composite model was generated using the workflow previously described in Mueller *et al*.^23^. This workflow integrates and prioritizes information from both a curated GSM model of a related organism and reviewed gene annotations to expedite the generation of a GSM model for the organism of interest. The *i*Syn731 model of *Synechocystis* PCC 6803^24^ was used as the reference GSM model for both organisms. This organism was selected using sequence alignments of the 16 s sequences of the cyanobacterial strains targeted in the genome comparisons using Clustal2.1^41^. This comparison revealed a similar percent identity between previously modeled cyanobacteria *Synechococcus* 7002 and *Synechocystis* 6803^24^,^26^ and *Synechococcus* 7942, resulting in the selection of our previously developed *i*Syn731 model of *Synechocystis* 6803^24^. The workflow uses homolog pairs between *Synechocystis* and the *Synechococcus* strains to identify which reactions should be transferred to the new GSM model. To identify homolog pairs a bidirectional BLAST search between each organism pair was performed using an e value cutoff of 10^−2^. A result was determined to be a hit if the alignment length was at least 75% of the length of both the query and target genes, and if the raw score was at least 25% of the self-hit score of the query gene. A homolog pair was identified if both genes had only each other as a hit^42^. This process was repeated to identify homologs between *Synechococcus* 7942 and 2973. Given that the organisms are closely related, the score cutoff was increased to 75% of the self-hit score. This reduced the number of occurrences of multiple hits for a gene and identified only the best hit, increasing the number of homolog pairs from 2274 to 2538. These homolog pairs were used to map reviewed annotations from *Synechococcus* 7942 to *Synechococcus* 2973. The composite model is available in both table (Supplementary File S1) and Systems Biology Markup Language (SBML) formats (Supplementary Files S2 and S3)^43^. For the comparison with current industrial strains the requirements for a BLAST hit were reduced in order to account for the greater phylogenetic distance between the species (i.e. 10% of self-hit score, 50% of length).

The biomass equation previously developed for the *i*Syn731 model was updated for the *Synechococcus* strains using experimental measurements for both strains. All biomass measurements for both strains were taken from Abernathy *et al*. (Abernathy *et al*., in review). These measurements were used to determine biomass equation stoichiometric coefficients for lipids, glycogen, amino acids, and chlorophyll. The coefficients for the unmeasured precursors were updated by scaling the coefficients from the *i*Syn731 biomass equation so that all biomass precursors added up to 1 g per gDW of biomass. This standardization facilitates comparison between the two strains.

### Experimental Measurements of Carbon Uptake

Experiemental measurements of the rate of carbon dioxide uptake were taken for both strains. Both *Synechococcus* strains were grown in a MC-1000 multicultivator (Photon Systems Instruments) in standard BG11 medium at 38 C, and supplemented with 3% CO_2_ to an OD_720_ of 1.0. *Synechococcus* 2973 was grown at 500 μE·m^−2^·s^−1^ light and 7942 was grown at 300 μE·m^−2^·s^−1^ light, the light intensities at which each strain achieved its maximal growth rate^19^, allowing for comparison of factors contributing to those optimal growth rates. Two 1 mL samples were sealed in 13 mL hungate tubes with rubber septa and sparged with 3% CO_2_ for 5 minutes. One tube for each strain was placed on its side on a shaker under their respective white LED illumination at 38 C for 1 hour after which time total CO_2_ was measured. The second tube was measured immediately. Total CO_2_ was calculated as the sum of the dissolved + gaseous CO_2_ within a sealed system. Total CO_2_ of the system was determined after injecting 100uL 10 N HCl into the tube to gas out dissolved CO_2_, followed by quantification of the headspace CO_2_ content on a HP 5980 gas chromatograph under the following conditions: porapak N column; temperature, 100 °C; carrier gas, helium at a flow rate of 40 ml min^−1^; and TCD detector. These uptake rates were given as umol CO_2 _mg^−1^ chl hr^−1^. Using the molecular weight and the biomass stoichiometry (mmol chl gDW^−1^) of chlorophyll A these rates were converted to the units of CO_2_ uptake flux within the model (mmol CO_2_ gDW^−1^ hr^−1^). The original measurements and their conversion to fluxes are in Supplemental File S5.

### GSM Model Implementation

FBA was performed as described by Orth *et al*.^27^ to determine maximum flux through the biomass equation. The carbon dioxide uptake fluxes calculated from the experimental data were used as constraints on the exchange flux for carbon dioxide. Flux variability analysis (FVA)^44^ was used to identify the flux ranges of each reaction by iteratively minimizing and maximizing the flux of a single reaction subject to the FBA constraints, along with constraints on biomass and photon uptake. In the context of the model, photons are treated as two separate metabolites consumed by photosystems 1 and 2. The minimum required photons to achieve a biomass level were determined by fixing biomass, retaining the FBA constraints, and minimizing the sum of the exchange fluxes for photons for photosystems 1 and 2.

In order to compare *in silico* and *in vivo* essentiality, the GPR for each reaction was reviewed upon the removal of each individual gene. If the GPR logic statement was no longer true (i.e. if the gene was an essential subunit of a protein complex or the gene did not have any homologs) then the reaction was not allowed to carry flux for the *in silico* mutant. If the model could not produce at least 10% of the wild-type biomass the mutation was classified as lethal. Gene knockouts were performed *in silico* for every gene in the model. The *in vivo* results were taken from those genes that were identified as essential or non-essential from the transposon library of *Synechococcus* 7942 published by Rubin *et al*.^31^. These results were categorized using the GrowMatch terminology, where either the *in silico* and *in vivo* results for growth or no growth match (i.e. GG and NGNG) or conflict (i.e. GNG and NGG)^30^.

## Additional Information

**How to cite this article**: Mueller, T. J. *et al*. Identifying the Metabolic Differences of a Fast-Growth Phenotype in *Synechococcus* UTEX 2973. *Sci. Rep.* **7**, 41569; doi: 10.1038/srep41569 (2017).

**Publisher's note:** Springer Nature remains neutral with regard to jurisdictional claims in published maps and institutional affiliations.

## Supplementary Material

Supplementary File S1

Supplementary File S2

Supplementary File S3

Supplementary File S4

Supplementary File S5

## Figures and Tables

**Table 1:** Compilation of ORFs with SNPs that are essential to GSM model reactions.

Locus Tag	Protein Annotation	Number of Reactions Gene is Essential For	Pathway containing reaction (s)
M744_01335	ATP synthase F_0_F_1_ subunit alpha	2	ATP synthesis
M744_03965	ABC-transporter substrate-binding protein	1	Adenine salvage
M744_06650	CTP synthetase	2	CTP synthesis
M744_06850	Chorismate mutase	1	Phenylalanine/Tyrosine biosynthesis
M744_11685	Anthranilate synthase, component I	2	Tryptophan biosynthesis
M744_12130	Long-chain-fatty-acid CoA ligase	1	Fatty acid metabolism
M744_12285	Glutamate synthase	1	Glutamate biosynthesis

**Table 2:** Number of cytochrome c oxidase operons in model cyanobacteria.

Organism	Obligate Photoautotroph?	Capable of Fixing Nitrogen?	Cyt C Oxidase operons present
*Anabaena* 29413	No	Yes	5
*Nostoc punctiforme*	No	Yes	4
*Anabaena* 7120	No	Yes	3
*Cyanothece* 51142	No	Yes	3
*Synechocystis* 6803	No	No	2
*Synechococcus* 7002	No	No	2
*Synechococcus* 7942	Yes	No	1
*Synechococcus* 6301	Yes	No	1
*Synechococcus* 6312	Yes	No	1
